# Association of Erythrocyte-Related Indices with Immune-Related Adverse Events and Survival of Lung Cancer Patients Receiving Immune Checkpoint Inhibitors

**DOI:** 10.3390/ph18091299

**Published:** 2025-08-29

**Authors:** Zhan Wang, Ting Zou, Chen-Wei Liao, Xiang-Ping Li, Zhao-Qian Liu, Ze-Fu Liu, Juan Chen

**Affiliations:** 1 Lung Cancer and Gastrointestinal Unit, Department of Medical Oncology, Hunan Cancer Hospital/The Affiliated Cancer Hospital of Xiangya School of Medicine, Central South University, Changsha 410031, China; wangzhan@hnca.org.cn; 2 Department of Pharmacy, National Institution of Drug Clinical Trial, Xiangya Hospital, Central South University, Changsha 410008, China; zouting@csu.edu.cn; 3 National Clinical Research Center for Geriatric Disorders, Xiangya Hospital, Central South University, Changsha 410008, China; xylxping@126.com (X.-P.L.); zqliu@csu.edu.cn (Z.-Q.L.); 4 Xiangya School of Pharmacy, Central South University, Changsha 410078, China; 19108483640@163.com; 5 Department of Urology, Xiangya Hospital, Central South University, Changsha 410008, China

**Keywords:** ICIs, irAEs, PFS, OS, best response, RBC, HCT, MCV

## Abstract

**Background**: Lung cancer has the highest lethality rate among malignancies worldwide. Immunotherapy is one of the common treatments for lung cancer patients. There are two main types of immunotherapies: one targets programmed cell death 1 (PD-1), and the other targets programmed cell death ligand 1 (PD-L1). These two belong to the class of immune checkpoint inhibitors (ICIs). However, immune-related adverse reactions (irAEs) were the main reasons affecting its clinical therapeutic effect. **Methods**: This retrospective cohort study analyzed red blood cell count (RBC), hematocrit (HCT), erythrocyte mean corpuscular volume (MCV) and immunotherapy outcomes in 920 lung cancer patients receiving immunotherapy from April 2019 to May 2023. **Results**: We found that high levels of RBC (>4.105 × 10^9^, *p* = 0.007, OR = 0.467, 95%CI: 0.268~0.812) and MCV (>86.35, *p* = 0.017, OR = 0.0.441, 95%CI: 0.224~0.865) were significantly related to the better response of ICIs immunotherapy in patients. Patients with high levels of HCT (>39.75%, *p* = 0.035, OR = 0.737, 95%CI: 0.555~0.979) may have a lower rate of irAEs occurrence. Meanwhile, patients with a low level of RBCs (≤4.635 × 10^9^, *p* < 0.001, OR = 1.636, 95%CI: 1.365~1.960) may have a longer period of PFS (progression-free survival), and patients with RBC (≤4.43 × 10^9^, *p* = 0.033, OR = 0.480, 95%CI: 0.244~0.941) may have a longer time of OS (overall survival). **Conclusions**: The findings indicate that the levels of RBC, MCV and HCT were significantly associated with the response and irAEs of ICIs in lung cancer patients. The levels of RBC might act as a possible biomarker for predicting the survival of lung cancer patients who are receiving ICI therapy.

## 1. Introduction

Lung cancer ranks as the top cause of cancer-associated deaths globally [1]. It is classified into different histologic subtypes, including non-small cell lung cancer (NSCLC) and small cell lung cancer (SCLC) [2]. At present, the commonly used treatment approaches include surgical removal, chemical therapy, radiation therapy, targeted therapy, and immunotherapy [3]. However, despite these options, the 5-year survival rate of lung cancer is still very poor [4]. For patients with advanced NSCLC, the combination of immunotherapy and chemotherapy serves as the first-line treatment, and it can enhance their survival outcomes. [5]. However, the occurrence of immune-related adverse reactions (irAEs) may affect the efficacy of immunotherapy [6]. irAEs are a range of adverse reactions after the treatment of ICIs [7]. Recently, more and more adverse immune reactions have been reported in lung cancer patients, such as cardiotoxicity, pneumonitis, dermal toxicity, gastrointestinal toxicity, colitis, hepatitis, endocrine, and musculoskeletal dysfunctions [8,9,10]. It seems that the irAEs may be related to the outcomes of ICIs, but the occurrence of adverse reactions and the efficacy of ICIs vary from person to person [11]. To find the biomarkers and identify patients who will benefit from immune checkpoint inhibitors (ICIs) is of great importance in cancer clinical treatment [12]. Thus far, most of the biomarker studies have been focused on tumor signatures such as the expression of PD-L1, the instability of microsatellites, and tumor genomic mutation; however, there are far fewer studies investigating clinical biomarkers [13]. The specific relevant indices are still being discovered along the way.

Two major classes of ICIs that are widely used are programmed cell death-1 (PD-1)/programmed death-ligand 1 (PD-L1) and cytotoxic T-lymphocyte antigen 4 (CTLA4) inhibitors [14]. The PD-1 antibody is one of the most studied and fastest growing immunotherapies in clinical practice [15]. Programmed cell death ligand 1 and 2 (PD-L1/L2) are expressed in antigen-presenting cells, while PD-L1 is additionally found in various tissues [16]. The interaction between PD-1 and PD-L1 triggers a co-inhibitory signal during T cell activation, which can suppress the cytotoxic function of T cells and exert a negative regulatory effect on the human immune response [17]. PD-L1 is highly expressed in tumor tissues, and it is capable of regulating the function of CD8+ T cells that infiltrate tumors [18]. PD-1/PD-L1 inhibitors can specifically bind to PD-L1 on tumor cells to inhibit its expression so that the function of the suppressed T cells can recover the recognition function of tumor cells so as to achieve an anti-cancer effect through the body’s own immune system [19]. Thus, immune regulation that targets the PD-1/PD-L1 pathway holds considerable importance in the battle against lung cancer [20]. It is well established that antibodies targeting PD-1, its ligand PD-L1, or cytotoxic T lymphocyte-associated protein-4 (CTLA-4) represent an advancing therapeutic approach for lung cancer. However, only a small proportion of patients gain advantages from this type of treatment [21]. Immune-related adverse events, known as irAEs, are usually mild in nature, can be addressed with proper treatment, and have the potential to reverse these effects. That said, on rare occasions, such side effects may become severe, which could necessitate stopping immunotherapy [22]. Therefore, it is crucial to find indicators related to ICIs’ efficacy and irAEs.

Blood routine is a routine test for lung cancer patients, which includes the neutrophil to lymphocyte ratio (NLR), hematocrit (HCT), white blood cell count (WBC), red blood cell count (RBC), mean corpuscular volume (MCV) and platelet to lymphocyte ratio (PLR) [23]. The occurrence, development and metastasis of tumors are inextricably related to peripheral blood [24]. The lymphocytes, neutrophils and platelets that we are familiar with in our usual blood tests all play a subtle role [25]. Lymphocytes are of great importance in inhibiting tumor cell proliferation and migration by inducing tumor cell apoptosis [26]. NLR and PLR have been reported to be significantly correlated with the prognosis of various malignant tumors such as NSCLC, melanoma, liver cancer and other forms of immunotherapy [27,28,29,30]. The NLR could potentially act as a useful predictive marker for irAEs and the survival results in NSCLC patients receiving PD-1 inhibitor therapy [31]. In the group of advanced NSCLC patients undergoing immunotherapy, individuals with a low PLR are inclined to have more positive OS and PFS [32]. In a mass study, RBC was shown to cause serious changes in reticulocytes and transient alterations in platelet counts [33]. The novel model composed of histologic subtypes, CD19+ B cells, regulatory T cells and RBC can predict the survival in nasopharyngeal carcinoma patients treated with ICIs [34]. We hypothesize that ERIs (erythrocyte-related indices) may be associated with the outcomes of immunotherapy in lung cancer patients.

## 2. Results

### 2.1. The Clinical Features of the Patients

The study details the clinical characteristics, irAES, and the best response observed in 920 individuals with lung cancer in Table 1. The general clinical data were analyzed and classified into the irAEs group, the non-irAEs group, the CR, PR, and SD group and the PD group. Overall, 804 patients received PD-1 immunotherapy as first-line treatment. Most of them were at an extensive stage when first diagnosed such as III or IV (732/920, 79.57%). A large proportion of the patients had a history of smoking (620/920, 67.39%). After statistical analysis, it was found that there were noteworthy differences between different groups regarding age and histology with irAE or best response. We collected the statistics of PFS and OS among 504 of them, which are shown in Table 2. The median duration for progression-free survival (MST-PFS) stood at 0.98 years, while the median overall survival (MST-OS) was 0.68 years.

### 2.2. The Association of ERIs with irAEs

**Figure 1:**
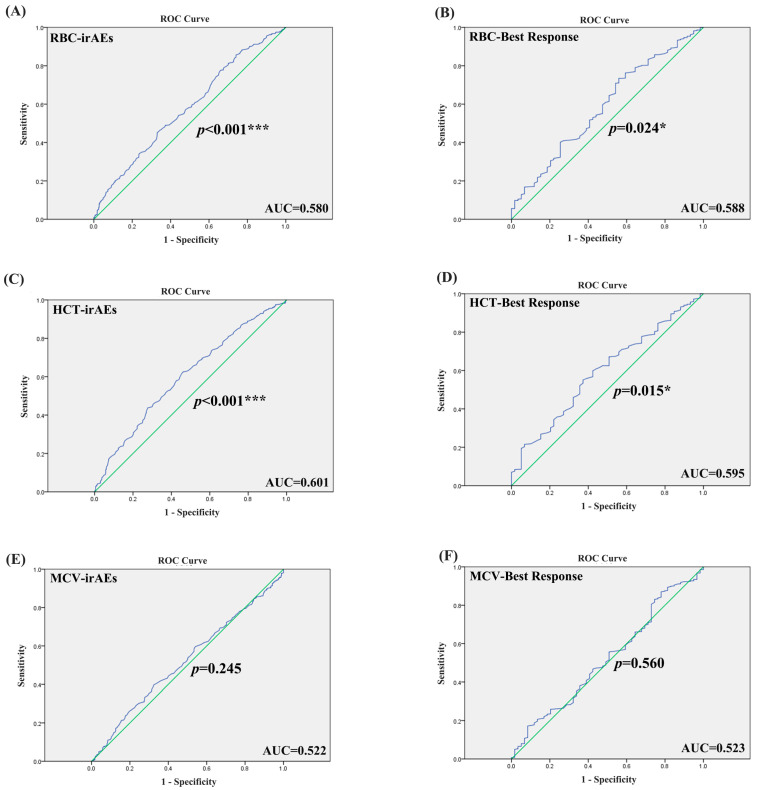
The ROC curve of ERIs with irAEs and best response. (**A**) The ROC curve of RBC with irAEs. (**B**) The ROC curve of RBC with best response. (**C**) The ROC curve of HCT with irAEs. (**D**) The ROC curve of HCT with best response. (**E**) The ROC curve of MCV with irAEs. (**F**) The ROC curve of MCV with best response. * *p* < 0.05, *** *p* < 0.001. Blue represents the ROC curve, and green represents that sensitivity + specificity = 1.

**Figure 2:**
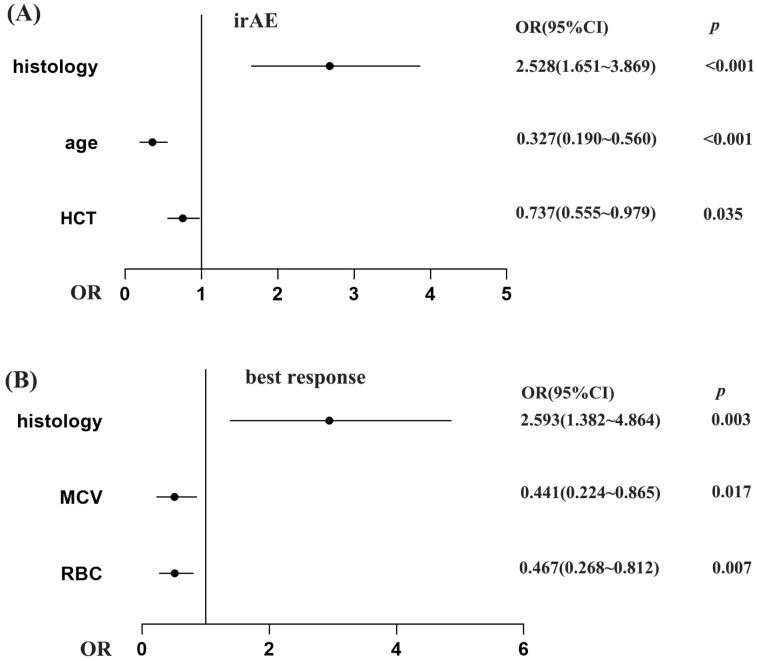
The association of ERIs with irAEs (**A**) and best response (**B**) in multivariate binary logistic regression. The x-axis reflects the OR value. Graphed by GraphPad Prism 9.

**Figure 3:**
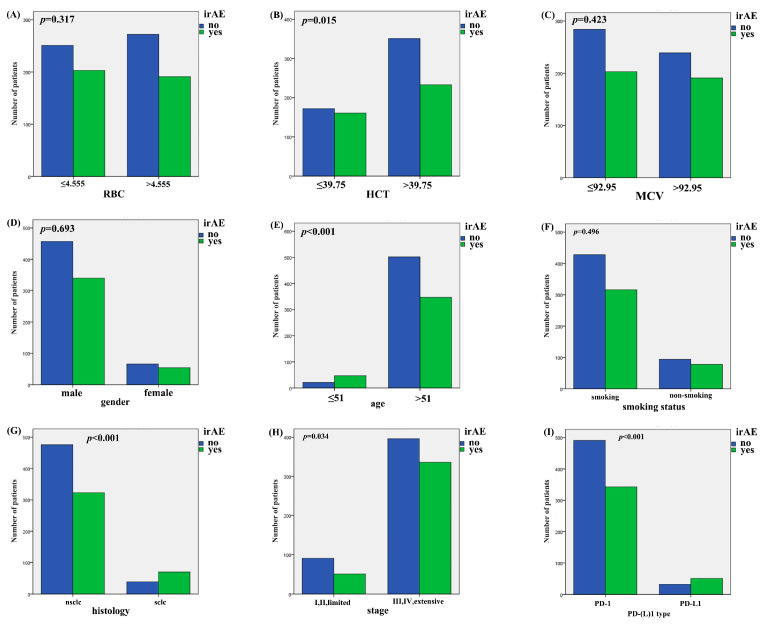
The association of ERIs and clinical characteristics with irAEs in χ2 test. (**A**) Association of irAEs with RBC, (**B**) HCT, (**C**) MCV, (**D**) gender, (**E**) age, (**F**) smoking status, (**G**) histology, (**H**) stage, and (**I**) PD-(L)1 type.

Patients with irAE or non-irAE have been divided into two groups. According to the irAE and ERIs of the 920 lung cancer patients, the ROC curve was used to determine the optimal cut-off value of ERIs. The cut-off values to define the high or low value of RBC, HCT and MCV were 4.555 × 10^9^ L, 39.75% and 92.95 fl, respectively, the AUC (area under the curve) values of the RBC, HCT and MCV were 0.580, 0.601 and 0.522, respectively, the *p* values of the RBC, HCT and MCV were *p* < 0.001, *p* < 0.001, and *p* = 0.245, respectively (Figure 1A,C,E). We used both multivariate binary logistic regression and the χ2 test to assess the association between the ERIs and irAEs. The results revealed that histology, age and HCT were significantly associated with irAEs in multivariate binary logistic regression (histology: *p* < 0.001, OR = 2.528, 95%CI: 1.651~3.869; age: *p* < 0.001, OR = 0.327, 95%CI: 0.190~0.560; HCT: *p* = 0.035, OR = 0.737, 95%CI: 0.555~0.979) (Figure 2A). That means that the possibility of irAEs in SCLC patients is 2.528 times that of NSCLC patients (OR = 2.528). Patients with age > 51 years are 0.327 times more likely to develop irAEs than those under 51 years old (OR = 0.327), while patients with HCT > 39.65% are 0.737 times more likely to develop irAEs than those with HCT < 39.65% (OR = 0.737). We also found that histology, age and HCT were significantly related to irAE in the χ2 test (histology: *p* < 0.001, age: *p* < 0.001, HCT: *p* = 0.015) (Figure 3B,E,G). The multivariate binary logistic regression results of irAEs are also shown in Table 3. However, the RBC and MCV do not show any significant correlation with irAEs both in multivariate binary logistic regression and the χ2 test.

### 2.3. The Association of ERIs with Immunotherapy Efficacy

**Figure 4:**
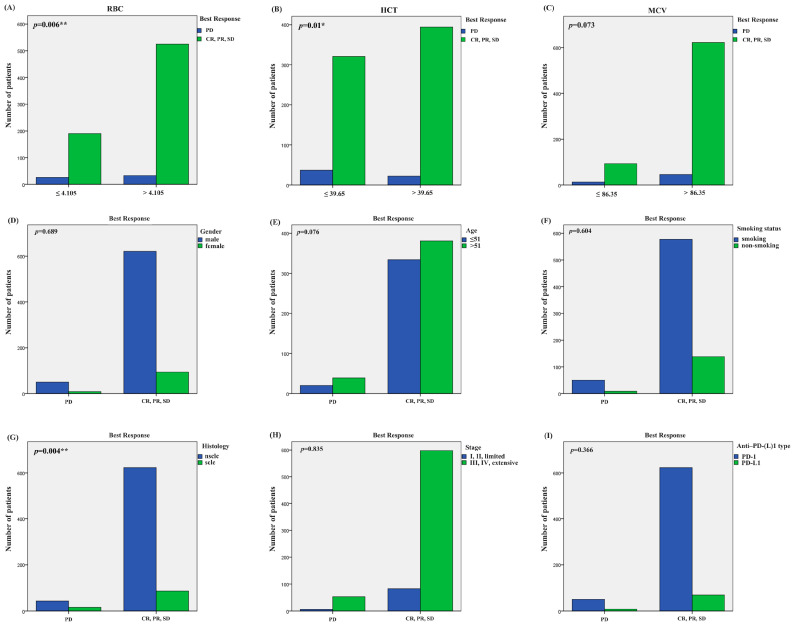
The association of ERIs and clinical characteristics with best response in χ2 test. (**A**) Association of best response with RBC, (**B**) HCT, (**C**) MCV, (**D**) gender, (**E**) age, (**F**) smoking status, (**G**) histology, (**H**) stage, and (**I**) PD-(L)1 type. * *p* < 0.05, ** *p* < 0.01.

We divided the best responses of the lung cancer patients into responders (CR: complete response, PR: partial response, SD: stable disease) and non-responders (PD: progressive disease). According to the best response and ERIs of the 920 lung cancer patients, we additionally employed the receiver operating characteristic (ROC) curve to identify the optimal threshold value for ERIs. The cut-off values to define the high or low value of the RBC, HCT and MCV were 4.105 × 109 L, 39.65% and 86.35 fl, respectively, the AUC values of the RBC, HCT and MCV were 0.588, 0.595 and 0.523, respectively, and the *p* values of the RBC, HCT and MCV were *p* = 0.024, *p* = 0.015, *p* = 0.560, respectively (Figure 1B,D,F). We also used multivariate binary logistic regression and the χ2 test to analyze the association between the ERIs and best response. We found that the histology, MCV and RBC are significantly associated with the best response in multivariate binary logistic regression (*p* = 0.003, OR = 2.593, 95%CI: 1.382~4.464; *p* = 0.017, OR = 0.441, 95%CI: 0.224~0.865; *p* = 0.007, OR = 0.467, 95%CI: 0.268~0.812) (Figure 2B). Therefore, the possibility of best response in PD in SCLC patients is 2.593 times that of the NSCLC patients (OR = 2.593), while the possibility of best response for patients with MCV > 86.35 is 0.441 times that for patients with MCV < 86.35 (OR = 0.441), and the possibility of best response for patients with RBC > 4.105 is 0.467 times that for patients with RBC < 4.105 (OR = 0.441). We also found that RBC, HCT and histology were significantly related to best response in the χ2 test (RBC: *p =* 0.006, HCT: *p =* 0.01, histology: *p* = 0.004) (Figure 4A,B,G). The multivariate binary logistic regression results of best response are also shown in Table 4.

### 2.4. The Association of ERIs with irAEs and Immunotherapy Efficacy in Student’s t-Test and Kruskal–Wallis Test

Firstly, we ran a normality test on RBC, HCT and MCV; only the RBC value conforms to normal distribution. We used Student’s *t*-test to analyze the association between RBC and irAEs or best response. It shows that RBC is significantly associated with the irAEs and best response (*p* < 0.001, *p* = 0.011, respectively). The HCT is significantly related to the irAEs and best response analyzed by the Kruskal–Wallis test (*p* < 0.001, *p* = 0.015, respectively). However, the MCV does not show any significant correlation with irAEs and best response in the Kruskal–Wallis test. The specific results are shown in Table 5.

### 2.5. The Relationship Between ERIs and Survival Outcomes in Lung Cancer Patients Undergoing Treatment with ICIs

**Figure 5:**
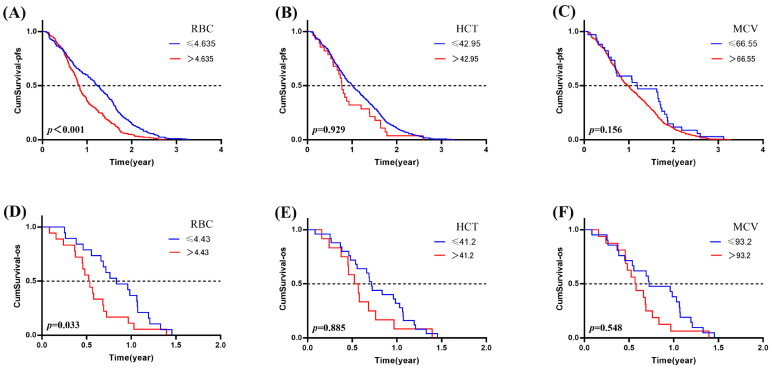
The association of ERIs with the prognosis in lung cancer patients treated with ICIs. The association of (**A**) RBC, (**B**) HCT and (**C**) MCV with PFS; the association of (**D**) RBC, (**E**) HCT, and (**F**) MCV with OS.

In our investigation, RBC showed a statistically significant association with PFS (*p* < 0.001) and OS (*p* = 0.031) among lung cancer patients treated with anti-PD-1/PD-L1 agents. This means that patients with a low count of RBC (≤4.635 × 10^9^) may have a better PFS than the patients who have a higher degree of RBC (*p* < 0.001, OR = 1.636, 95%CI: 1.365~1.960). Patients with a low count of RBC (≤4.43 × 10^9^) may have a better OS than the patients who have a higher degree of RBC (*p* = 0.033, OR = 0.480, 95%CI: 0.244~0.941). To sum up, a lower level of RBC appears to have a protective effect on the prognosis of lung cancer patients when treated with PD-1 or PD-L1 inhibitors (Figure 5).

## 3. Discussion

There are various systemic treatments including immunotherapy, chemotherapy, radiation therapy, surgical resection, cellular therapy and targeted therapy in lung cancer treatment [35]. Immunotherapies, which encompass ICIs, have emerged as the central therapeutic approach for lung cancer patients suffering from advanced or metastatic conditions [36]. Recently, immunotherapy has also been moved into early-stage lung cancer treatment, which had few treatment advances in the past few years [37]. A novel domain in the realm of cancer therapy has been unlocked by immunotherapy, which leverages the body’s inherent immune system to combat tumor cells [38]. The immune response against tumor cells can be stimulated by ICI target receptors like PD-(L)1 and CTLA-4 [39]. Nonetheless, a multitude of elements continue to exert an influence on how well immunotherapy performs [40]. The sensitivity of immunotherapy can be modulated by different pro-immunogenic or immunosuppressive features in the tumor microenvironment [41]. The responsiveness to ICIs and the incidence of irAEs could influence the therapeutic outcomes in lung cancer patients [42]. Therefore, it is crucial to find the indicators that affect the effectiveness of immunotherapy treatment.

In this study, we found that ERIs RBC, HCT and MCV play important roles in irAEs and immunotherapy efficacy. Overall, 43.15% of the patients in our study suffered from irAEs (397/920). HCT is significantly associated with the irAEs (*p* = 0.035, OR = 0.737, 95%CI: 0.555~0.979) in both multivariate binary logistic regression and the χ2 test (*p* = 0.015). Research exploring the association between HCT and immunotherapy in lung cancer remains limited. As demonstrated by Salman et al., PD-1-treated hepatocellular carcinoma mice showed no meaningful correlation with body weight, activity patterns, physical appearance, or hematocrit levels [43]. Korsen et al. found that platelets and HCT transiently dropped, reaching nadir at 2 to 3 weeks in NEPC (neuroendocrine prostate cancer) mice treated by a radio-immunotherapeutic agent [44]. It has been reported that there are statistical differences in several blood routine indices: the HCT, hemoglobin and lymphocyte ratio decreased after thermal ablation combined with AFK cells immunotherapy in patients with malignancies [45].

Overall, 77.71% (715/920) of the patients in our study were classified as having one of the best responses (CR, PR and SD). The RBC and MCV were found to be significantly correlated with the best response analyzed by multivariate binary logistic regression (*p* = 0.007, OR = 0.467, 95%CI: 0.268~0.812; *p* = 0.017, OR = 0.441, 95%CI: 0.224~0.865; respectively). The RBC and HCT are significantly associated with best response in the χ2 test (*p* = 0.006, *p* = 0.01; respectively). It has been reported that red blood cell (RBC) transfusions can lead to iron overload, which may increase the risk of end-organ complications in patients with anemia [46]. Certain parameters may influence the therapeutic outcomes in lung cancer patients. Research conducted by Wang et al. demonstrated that while CD47/SIRPα signaling can enhance innate immune responses, CD47-mediated effects might simultaneously trigger red blood cell toxicity, potentially compromising immunotherapy effectiveness [47].

It has been reported by Ming Wu et al. that the red blood cell (RBC)-hitchhiking strategy can deliver the neoantigen DNA vaccine to targeted hepatocellular carcinoma (HCC) immunotherapy, which can drive personalized antitumor immunity for HCC [48]. RBC can reduce tumor hypoxia and induce oxidative damage against tumor cells, which may act as a potential candidate in cancer therapy [49]. The OS benefit treated by ICIs was associated with higher levels of MCV, MPV, erythrocytes, lymphocytes and hemoglobin, which means MCV may be a potential biomarker in NSCLC patients on ICIs [50]. It has also been reported that the red cell-based score on PFS and OS may have a prognostic impact on the immunotherapy efficacy in patients with metastatic renal cell carcinoma [51].

It has been reported that when the body has an inflammatory response, the homeostasis process may be disrupted, which can lead to acute or chronic anemia and then subsequently have an effect on RBC/HCT/MCV [52]. Inflammation can directly cause the destruction of red blood cells, which leads to a low level of RBC/HCT/MCV [53]. It has also been reported that inflammation can predict the prognosis of ICIs in pancreatic ductal adenocarcinoma [54]. Inflammation may affect ERIs and thereby influence the effectiveness of immunotherapy. It would be valuable for future studies to research the ability of inflammation on ERIs to affect ICIs prognosis.

It seems paradoxical that elevated red blood cell parameters are correlated with a more favorable response to ICIs but at the same time with lower survival (OS and PFS). We should specify that the best response and survival have no necessary connection. The cut-off value with RBC and best response was 4.105 × 10^9^, while the RBC and survival (PFS) values were 4.635 × 10^9^ and 4.43 × 10^9^ (OS).

Our research has a number of limitations. Firstly, it adopted a retrospective observational design, and the follow-up of patients depends on the electronic medical record. Secondly, the sample was not large enough. As we performed the correction for multiple comparisons, which calculated the *p* value of the variables by Bonferroni correction (FWER), no significant ERIs remained, but in Holm–Bonferroni correction (FWER), the corrected *p* value of the RBC was still positive with the best response. Finally, validation by independent samples is needed. Future investigations will incorporate expanded clinical datasets to evaluate the prognostic significance of erythrocyte parameters in immunotherapy outcomes. We will also conduct research at animal and cellular levels. The results we found in our study are very important for the clinical guidance in the use of PD-1/PD-L1 immunotherapy. RBC could serve as a potential biomarker for predicting the prognosis of lung cancer patients undergoing treatment with ICIs.

## 4. Patients and Methods

### 4.1. Patient Collection

All participants included in our research were chosen based on the following criteria: (1) individuals who received their initial diagnosis of lung cancer at either Xiangya Hospital of Central South University or Hunan Cancer Hospital (located in Changsha, Hunan Province, China) between April 2019 and May 2023; (2) patients who received PD-1 or PD-L1 immunotherapy; (3) all disease stages determined in accordance with the eighth version of the TNM staging system [55]; (4) irAEs categorized based on the National Cancer Institute’s Common Terminology Criteria for Adverse Events, version 5.0 [56]. irAEs were caused by immune dysfunction which may require frequent monitoring or even require the use of immunosuppressive or endocrine replacement therapy according to NCCN and CSCO guidelines [57]. This retrospective study was approved by the ethics committee of Xiangya Hospital, Central South University in 15 April 2021 (2022100970).

### 4.2. Treatment and Data Collection

The clinical features of the patients were gathered, including age, sex, smoking history, disease stage, histological type, type of anti–PD-(L)1 therapy, best response, and irAEs. We additionally examined the relationship between ERIs and the prognostic outcomes of lung cancer patients with the relevant findings compiled in Table 2. The ERIs (including RBC, HCT, MCV) of the enrolled patients before the first treatment of ICIs were obtained from the medical records. The best treatment response was evaluated by the oncologist.

### 4.3. Statistical Analysis

In the present research, measured data were presented in the form of individual cases, while variables belonging to categorical types were indicated by means of frequencies and the corresponding percentages within each group. To identify discrepancies, comparisons of data across different groups were conducted utilizing either Student’s *t*-test or the Kruskal–Wallis test. Multivariate binary logistic regression and the χ2 test were used to analyze the correlations of independent factors associated with irAEs or clinical efficacy. We further employed Cox proportional hazard models to assess variations in variables such as ERIs, histological type, age, clinical stage, smoking history, sex, and metastasis between PFS and OS. The forward stepwise approach within Cox proportional hazard models was utilized by us to identify the covariates. The *p* value was two-sided, and *p* < 0.05 was considered statistically significant. The cut-off value of the parameter was determined by the ROC curve and calculating the Youden exponent. All the statistical analyses mentioned above were carried out with the help of SPSS 18.0 (developed by SPSS Inc., located in Chicago, IL, USA) and GraphPad Prism (version 9, https://www.graphpad.com/). The flow chart is shown in Figure S1.

## 5. Conclusions

To sum up, the findings of our research suggest that the ERIs including RBC, HCT, and MCV could serve as predictors for the onset of irAEs and the prognostic outcomes of lung cancer patients receiving ICI therapy.

## Figures and Tables

**Table 1:** Baseline clinical features of the 920 lung cancer patients treated with anti-PD (L)1 immunotherapy.

Characteristic	irAEs, No. (%)		
	None	Single	Multiple
No.	523	166	231
Age, median (range), y	49 (34–80)	55 (34–75)	63 (38–83)
Gender			
Male	457 (87.4)	145 (86.8)	198 (85.7)
Female	66 (12.6)	21 (12.7)	33 (14.3)
Smoking status			
Ever	428 (81.8)	141 (84.9)	178 (77.1)
Never	94 (18.0)	25 (15.1)	53 (22.9)
NA	1 (0.2)	0 (0.0)	0 (0.0)
Stage at diagnosis			
I, II, limited	78 (16.5)	46 (28.8)	8 (3.5)
III, IV, extensive	396 (83.5)	114 (71.2)	222 (96.5)
Histology			
Adenocarcinoma	150 (28.7)	49 (29.5)	84 (36.4)
Squamous cell	323 (61.8)	91 (54.8)	101 (43.7)
NOS	50 (9.6)	26 (15.7)	46 (19.9)
Anti-PD-(L)1 type			
PD-1	460 (93.5)	134 (81.7)	210 (90.9)
PD-L1	32 (6.5)	30 (18.3)	21 (9.1)
irAEs to first therapy, median (range), wk	NA	11 (0–133.71)	3 (0–80.43)
Best treatment response			
CR, PR, SD	383 (73.2)	129 (77.7)	203 (87.9)
PD	21 (4.0)	16 (9.6)	22 (9.5)
NA	119 (22.8)	21 (12.7)	6 (2.6)

PD-1: Pembrolizumab, Camrelizumab, Sintilimab, Toripalimab, Nivolumab, Tislelizumab, Serplulimab, Penpulimab. PD-L1: Atezolizumab, Durvalumab, Sugemalimab.

**Table 2:** Distribution of characteristics in lung cancer patients and prognosis analysis.

Variables	Patients N (%)	Death N (%)	MST-PFS (Year)	MST-OS (Year)
Lung cancer	504	37	0.98	0.68
NSCLC	403 (80.0)	24 (64.9)	1.07	0.69
SCLC	101 (20.0)	13 (35.1)	0.75	0.52
Age				
≤62	261 (51.8)	13 (35.1)	1.02	0.68
>62	243(48.2)	24 (64.9)	0.95	0.63
Clinical stage				
I/II/LD	17 (3.4)	0 (0)	1.30	NA
III/IV/ED	487 (96.6)	37 (100.0)	0.98	0.68
Smoking status				
Non-smoker	113 (22.4)	31 (83.8)	1.04	0.52
Smoker	391 (77.6)	6 (16.2)	0.98	0.69
Gender				
Male	429 (85.1)	32 (86.5)	0.99	0.68
Female	75 (14.9)	5 (13.5)	0.98	0.66
Metastasis				
PD-1	443 (87.9)	33 (89.2)	0.67	0.68
PD-L1	61 (12.1)	4 (10.8)	1.06	0.93

**Table 3:** The association between characteristics with irAEs in univariate binary logistic regression.

Factors	Beta	*p* Value	OR (95%CI)
Histology	0.927	<0.001	2.528 (1.651~3.869)
age	−1.119	<0.001	0.327 (0.190~0.560)
HCT	−0.305	0.035	0.737 (0.555~0.979)
constant	0.893		

**Table 4:** The association between characteristics with best response in multivariate binary logistic regression.

Factors	Beta	*p* Value	OR (95%CI)
Histology	0.953	0.003	2.593 (1.382, 4.864)
RBC	−0.762	0.007	0.467 (0.268~0.812)
MCV	−0.820	0.017	0.441 (0.224~0.865)
constant	−1.464		

**Table 5:** The association between RBC, HCT and MCV with irAEs and best response in Student’s *t*-test and Kruskal–Wallis test.

Factors	Methods	*p* Value	
		irAEs	Best Response
RBC	Student’s *t*-test	<0.001	0.011
HCT	Kruskal–Wallis test	<0.001	0.015
MCV	Kruskal–Wallis test	0.232	0.560

## Data Availability

The original contributions presented in this study are included in the article/Supplementary Material. Further inquiries can be directed to the corresponding authors.

## Supplementary Materials

The following supporting information can be downloaded at: https://www.mdpi.com/article/10.3390/ph18091299/s1, Figure S1: Flow chart of our study.

## Abbreviations

AUC	area under curve
PD-1	programmed cell death 1
PD-L1	programmed cell death ligand 1
ICIs	immune checkpoint inhibitors
irAEs	immune-related adverse reactions
RBC	red blood cell count
HCT	hematocrit
MCV	erythrocyte mean corpuscular volume
ERIs	erythrocyte related indices
NSCLC	non-small cell lung cancer
SCLC	small cell lung cancer
CTLA-4	cytotoxic T lymphocyte-associated protein-4
NLR	neutrophil to lymphocyte
PLR	platelet to lymphocyte
CR	complete response
PR	partial response
SD	stable disease
PD	progressive disease
OS	overall survival
PFS	progression-free survival
MST	median survival time
