# Supplementary figures and images for: Association of Erythrocyte-Related Indices with Immune-Related Adverse Events and Survival of Lung Cancer Patients Receiving Immune Checkpoint Inhibitors
Source: Pharmaceuticals (Basel). 2025 Aug 29;18(9):1299. doi: 10.3390/ph18091299 (PMC12472678; doi:10.3390/ph18091299)

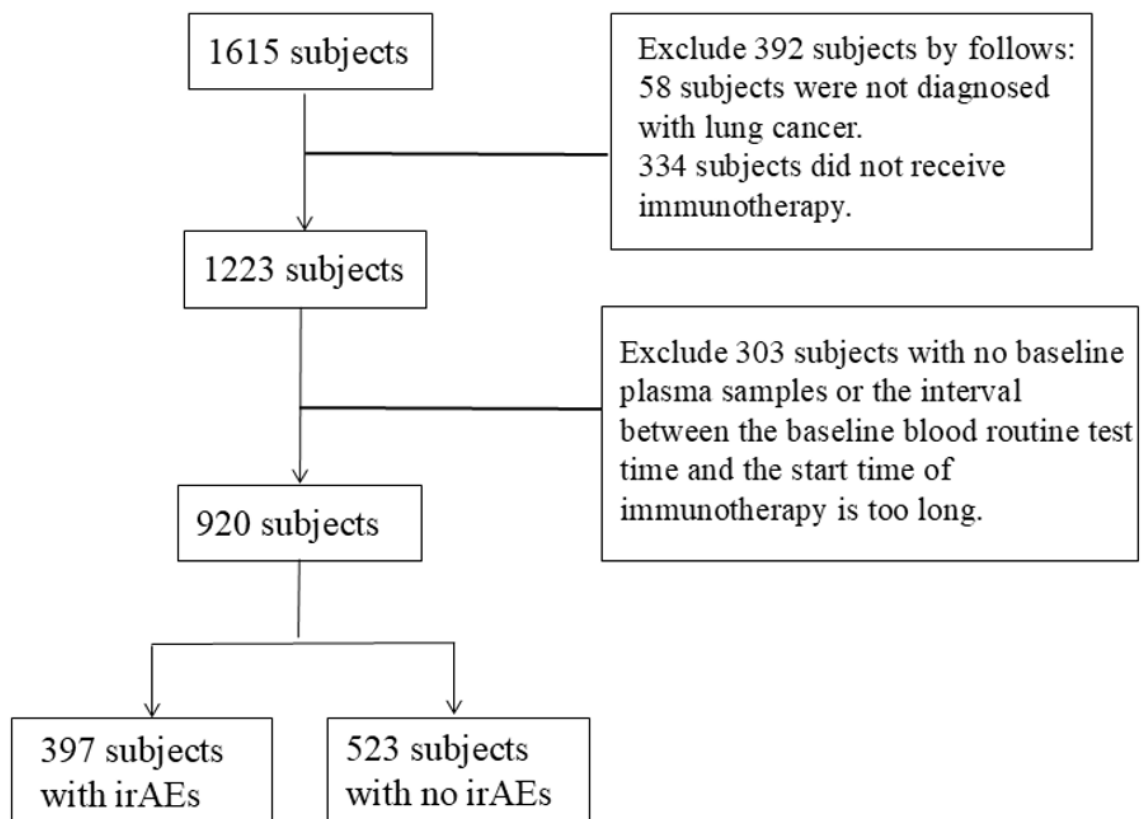

**Figure S1.** Flow chart of our study.

Supplement: Supplementary file 1 [file pharmaceuticals-18-01299-s001.zip › pharmaceuticals-3788641-supplementary.pdf]
